# Antioxidant activity and ultrastructural changes in gastric cancer cell lines induced by Northeastern Thai edible folk plant extracts

**DOI:** 10.1186/1472-6882-13-60

**Published:** 2013-03-13

**Authors:** Praphaporn Stewart, Patcharee Boonsiri, Songchan Puthong, Panadda Rojpibulstit

**Affiliations:** 1 Division of Anatomy and Department of Preclinical Science, Faculty of Medicine, Thammasat University, Pathumthani, Thailand; 2 Department of Biochemistry, Faculty of Medicine, Khon Kaen University, Khon Kaen, Thailand; 3 Institute of Biotechnology and Genetic engineering, Chulalongkorn University, Bangkok, Thailand; 4 Division of Biochemistry, Department of Preclinical Science, Faculty of Medicine, Thammasat University, Pathumthani, Thailand

**Keywords:** Gastric cancer, Ultrastructure, Cell cytotoxicity, TEM

## Abstract

**Background:**

Phytochemical products have a critical role in the drug discovery process. This promising possibility, however, necessitates the need to confirm their scientific verification before use. Hence, this study aims to evaluate (1) the antioxidant activity, (2) cytotoxicity potential, and (3) the effect on ultrastructural alteration in gastric cancer cell lines through exposure to fractions of three local Northeastern Thai edible plants.

**Methods:**

Plants, *Syzygium gratum, Justicia gangetica* and *Limnocharis flava* were extracted with ethyl acetate, and each crude extract analysed for their total phenolics content by Folin-Ciocalteu method. Their antioxidant activity was assessed using the ABTS system. The extracts were then assayed for cytotoxicity on two gastric cancer cell lines Kato-III and NUGC-4, and compared with Hs27 fibroblasts as a control using the MTT assay. The cell viability (%), IC_50_ values, as well as the ultrastructural alterations were evaluated after treatment with one way analysis of variance (ANOVA).

**Results:**

The total phenolic values of the ethyl acetate extracts were well correlated with the antioxidant capacity, with extracted product of *S. gratum* displaying the highest level of antioxidant activity (a 10-fold greater response) over *J. gangetica* and *L. flava* respectively. Exposure of *S. gratum* and *J. gangetica* extracts to normal cell lines (Hs27) resulted in marginal cytotoxicity effects. However, through a dose-dependent assay *S. gratum* and *J. gangetica* extracts produced cytotoxicological effects in just over 75 percent of Kato-III and NUGC-4 cell lines. In addition, apoptotic characteristic was shown under TEM in both cancer cell lines with these two extracts, whereas characteristics of autophagy was found in cell lines after post exposure to extracts from *L. flava*.

**Conclusions:**

From these three plants, *S. gratum* had the highest contents of phenolic compounds and antioxidant capacity. All of them found to contain compound(s) with cytotoxicity *in vitro* on cancer cells but not on normal cell lines as resolved in tissue culture and ultrastructural analysis. This is the first report to show the effect on cellular alteration as apoptosis of an ethyl acetate extract of *S. gratum* and *J. gangetica.* Further studies are now focused on individual isolates and their function, prioritizing on *S. gratum* and *J. gangetica* for the development of novel therapeutics and combatants against cancer.

## Background

Gastric cancer is the fourth most frequently diagnosed cancer and the second most leading cause of cancer related death in the world [1]. It was estimated that there were about 1 million new gastric cancer cases recorded in 2008 but of those, the majority (713,900) were reported in developing countries, with the highest incidences for gastric cancer found in Eastern Asia, over Central and Eastern Europe, and South America [1]. Despite the seeming intensification of the disease, evidence suggests that overall gastric cancer rates is bucking the trend, with a decrease in reports of gastric cancer found in most parts of the western world [2].

Unfortunately most gastric cancer patients are often diagnosed at an advanced stage when a cure is not possible and treatment is palliative with the intent of improving the quality and quantity of life. Even though, there are treatment guidelines for gastric cancer, the five-year survival rate is less than 50% [3,4]; a rate that is obviously not encouraging to either oncologists or cancer sufferers. In addition, side effects from current treatments i.e. surgery, chemotherapy and radiation are not satisfactory. So, targeted therapies are needed to decrease side effects and improve the clinical outcomes of patients. Hence, researchers in this new millennium are paying much more attention to the development of not only new therapeutic guidelines, and early prevention strategies for gastric cancer [5,6], but also on finding novel and target specific therapeutic agents as well.

Over the last two decades, phytochemical products have played a dominant role in the discovery of new drugs to target cancer [7], with over 60% of currently used anti-cancer agents are derived from natural sources [8]. Examples of worldwide clinically useful antitumor agents derived from wild plants include taxol, vinblastine, vincristine, camptothecin derivatives, topotecan (a wheat grass), sea-buckthorn, lingzhi, irinotecan, and etoposide, which is derived from epipodophyllotoxin [9,10]. Others are derived from fruits and vegetables; not limited to include curcumin (turmeric), genistein (soybean), catechins (green tea) [7], but also herbs like vinca alkaloids, podophyllotoxin, berberine, lemons grass oils, flavonoid and camptothecin; another group of promising anticancer agents [11]. Although these anti-cancer agents have been employed for targeted mechanism-based pathways, their effective manipulation of the extrinsic and the intrinsic apoptosis pathways are still being explored [12-14]. Paclitaxel isolate from the bark of the Pacific yew, *Taxus brevifolia* is one phytochemical that shows promise. It is a drug approved from the FDA to be used to treat AIDS-related Kaposi sarcoma, breast cancer, non-small cell lung cancer and ovarian cancer. Its primary cellular effect is to cause abnormal stabilization of the dynamic microtubule polymerization, leading to the failure of cell division resulting in apoptosis [15-17]. However, paclitaxel is also being studied as an alternative treatment for other types of cancer including gastric cancer. It is currently in clinical trials phase III [18,19]. Regardless of whether they have been approved or not, the broad reaching support and continuation of studies of plant extracts with implications in gastric cancer treatment are indicative of the continued role that natural products play in the drug discovery process.

When considering an epidemiology study of newly diagnosed gastric cancer cases in Thailand, much lower incidences have been observed in the Northeastern region [20]. Though, the prevalence of *Helicobacter pylori* infections, are not different across the northern region of Thailand, no geographical factor (for example plateau, mountainous range or jungle terrain) was different either [21]. Therefore, there must be something else central to the population in the Northeastern region that reduces the overall incidences of gastric cancer. Having all but ruled out genetics, and environmental factors, it has been suggested that edible folk plants diets that are usually consumed in this region, may hold the answer to this discrepancy. This new data, and this promising lead greatly challenges us to explore the mysterious phenomenon, that is whether the phytochemical compounds within Northeastern Thai edible folk plants have chemopreventive or cytotoxic potential to combat gastric cancer, or other properties. For that reason, this study therefore was undertaken to evaluate the cytotoxicity potential of these local edible plants. Of particular interest, plants *S. gratum*, *J. gangetica* and *L. flava*, were selected based on epidemiological data that suggests that they are the most regularly edible folk vegetables in the Northeastern region.

We postulate that these plants could hold within hidden properties that could be exploited to combat this cancer, and the present study seeks to assess their potential. We assess the crude phenolic-based extracts of these plants, and demonstrate high cellular apoptotic and cytotoxic effects in two common, and comparative gastric cancer cell lines, Kato-III and NUGC-4.

## Methods

### Plant materials

Three local edible folk plants; *S. gratum*, *J. gangetica* and *L. flava* (Table 1) were purchased from three different local markets in Khon Kaen province in the Northeastern part of Thailand during October to December 2008. These plants were selected based on ethnobotanical information [22-26] and epidemiological data as described above. Correct taxonomic identification of plant species used for this study was overseen by botanists from the Department of Botany and Pharmacology, Faculty of Pharmacy, Khon Kaen University, Thailand.

**Table 1:** The names of three plant extracts and other research references, therapeutic use in Thai traditional medicine screened in this study

**Species [Voucher number]**	**Family (Common name English/Thai)**	**Reported major constituents**	**Therapeutic use in Thai traditional medicine**	**Edible part**	**Ref.**
*Syzygium gratum* (Wight) S.N. Mitra var. *Gratum* [Ch. Laongpol 6]^a,c^	Myrtaceae (Eugenia/Phak Mek, Samet chun)	Not yet clearly determined in chemical structure but proved to be strong in antioxidants and the prevention of oxidative and nitrosative stresses	Treatment of dyspepsia and indigestion	Leaves	22,23
*Justicia gangetica* L. [TK-PSKKU-0066]^b^	Acanthaceae (Chinese violet, tropical primrose/Accepted name: *Asystasia gangetica* )	5,11-epoxymegastigmane glucoside (asysgangoside), salidroside, benzyl β-d-glucopyranoside, (6*S*,9*R*)-roseoside, ajugol, apigenin 7-*O*-β-d-glucopyranoside, apigenin 7-*O*-neohesperidoside, and apigenin 7-*O*-β-d-glucopyranosyl (1→6)-β-d-glucopyranoside	Treatment of stomach pain, stomach worms, anti-asthma	Leaves	24,25
*Limnocharis flava* L. Buchenau [Patt. 173]^c^	Limnocharitaceae (yellow velvetleaf, yellow burr head/Talabhat reusi)	Undetermined	Appetizer	Stem	26

^a^Voucher specimens deposited at the Forest Herbarium (BKF), Department of National Park, Wildlife and Plant Conservation, Ministry of Natural Resource, ^b^the Herbarium of the Faculty of Pharmaceutical Sciences, Khon Kaen University and ^c^the Prince of Songkla University herbarium (PSU), Department of Biology, Faculty of Science, Prince of Songkla University, Thailand.

### Preparation of plant extracts

Edible parts of each individual plant variety (Table 1) were rinsed with sterile distilled water to remove detritus and dried in hot air oven at 50°C for 7 days. Once dried, plant parts were then cut into small pieces and ground to a fine powder using a mortar and pestle. Each ground powder plant material was then immersed with the excess of the ethyl acetate solvent (Sigma-Aldrich Pte-Ltd, Singapore) in an extraction bottle. The ethyl acetate mixtures were then incubated on a shaker incubator at room temperature for 72 h. Following this process, the supernatants were then transferred to a new container, and the extraction process with ethyl acetate was repeated three more times, before the supernatants of these triplicate extractions were combined. These were then filtered through whatman filter paper no.1, and evaporated by a rotary evaporator. These sample extracts were then employed in further experiments.

### Determination of total phenolic compound

Total phenolic compounds in the plant extracts were determined by Folin-Ciocalteu method as described by Sachindra [27]. In brief, 0.2 mL each plant extract dissolved in 50% DMSO (Santa cruz biotechnology Inc., Bangkok, Thailand) was oxidized with 1.0 mL 10-fold-diluted Folin–Ciocalteu reagent (Sigma-Aldrich Pte-Ltd, Singapore) and neutralized with 0.8 mL of 6% sodium carbonate solution (Sigma-Aldrich Pte-Ltd, Singapore). After 1 h incubation, the absorbance of the solution was measured at 764 nm and the results were represented as milligram gallic acid equivalent per gram of dry weight (mg GAE/g). The assay was conducted in triplicate for each sample concentration from 3 separated assays.

### Determination of antioxidant activity

Antioxidant activity of the plant extracts was determined spectrophotometrically using the ABTS system according to the method of Re and colleagues [28]. Briefly, ABTS radical cation (ABTS^•+^) mixture was generated by the oxidation of 7 mM ABTS (Sigma-Aldrich Pte-Ltd, Singapore) with 140 mM potassium persulphate (Sigma-Aldrich Pte-Ltd, Singapore), incubated for 16 h at room temperature in the dark. Antioxidant activity was determined by adding 0.2 mL of plant extracts with 1.8 mL ABTS^•+^ radical cation mixture. After incubating the mixture for 6 min, the absorbance at 734 nm was recorded. ABTS^•+^ radical scavenging ability (%) of plant extracts were calculated based on the following equation: ABTS^•+^ radical scavenging ability (%) = [(Abs.control–Abs.test sample) /Abs.control] x100. Where Abs.control is the absorbance of control reaction (without plant extract) and Abs.test sample is the absorbance in the presence of a plant extract. The results were then compared to the anti-scavenging activity of Trolox (Sigma-Aldrich Pte-Ltd, Singapore) and represented as Trolox equivalent antioxidant capacity per gram of dry weight (TEAC/g). The assay was conducted in triplicate for each sample concentration from 3 separated assays.

### Cell culture

Two human gastric carcinoma cell lines Kato-III (ATCC No. HTB-103) from American Type Culture Collection (ATCC, Rockville, MD, USA) and NUGC-4 (JCRB0834) from the Health Science Research Resources Bank (Japan Health Sciences Foundation) were used for *in vitro* cytotoxic assays. The human foreskin fibroblast cell line Hs27 (ATCC No.1634) was used as a control. They were cultured in sterile RPMI 1640 containing 10% (v/v) fetal bovine serum (Biochrom AG, Berlin) at 37°C supplied with 5% CO_2_ in an incubator. Cells were grown in standard tissue culture flasks and upon reaching 80% confluence were passaged with a solution of 0.25% trypsin-EDTA (Sigma-Aldrich Pte-Ltd, Singapore) every 3–4 days until use.

### *In vitro* cytotoxicity assay

Plant extracts were assessed its cytotoxic activity against Kato-III and NUGC-4 cell lines via the MTT colorimetric assay as first described by Mosmann [29] with modifications suggested by Denizot and Lang [30]. Cultured cells (1 × 10^4^ cells) in complete media were transferred into each well of a flat 96 well plate and then incubated at 37°C in a humidified air atmosphere enriched with 5% (v/v) CO_2_ for 24 h in order to let the cells attach to the bottom of each well. The cultured cells were then treated with the tested crude extract (triplicate wells per condition) by the addition of 2 μL of serial dilutions of each extract at a concentration of 1.25, 2.5, 5, 10 and 20 μg/mL. The cells were then cultured as above for another 72 h prior to the addition of 10 μL of a 5 mg/mL solution of 3-(4, 5-dimethylthiazol-2-yl)-2, 5-diphenyltetrazolium bromide (MTT) (Sigma-Aldrich Pte-Ltd, Singapore) into each well. The incubation was continued for another 4 h before the media was removed. A mixture of DMSO (150 μL) and glycine (25 μL) was added to each well and mixed to ensure cell lysis and dissolving of the formasan crystals, before the absorbance at 540 nm was measured. Three replications of each experiment were performed and the percentage of MTT conversion to its formazan derivative for each well (percent cell growth) was calculated by dividing the OD at 540 nm of the wells with the control based on the following equation: Percent cell growth = [A540 test – A540 zero] × 100/[A540 control – A540 zero]. Where A540 zero = A540 of solution after the cell was incubated for 24 h before the addition of plant extracts; A540 test = A540 of solution after plant extracts addition; and A540 control = A540 of solution without plant extracts addition. In addition, for non-toxic assurance of plant extracts against normal cells (fibroblast cell line Hs27), a double dose (2 times of IC_50_ concentrations [10 μg/ml]) of the extracts were employed and assessed by MTT assay. The assay was conducted in triplicate for each sample concentration from 3 separated assays.

### Half maximal inhibitory concentration (IC_50_)

The obtained absorbance at 540 nm was used to determine the percentage of cell survival assuming that 100% survival was obtained when treated with solvents only as controls, and that no differences in metabolic activity existed between surviving cells under differing conditions. Under these assumptions, the percentage survival of the treated cancer cell lines and normal cultured cells was calculated according to the following formula: Percentage of survival = (A540 treated cells/A540 control) × 100. The mean ± 1 standard deviation (SD) cell survival (%) was plotted against the corresponding plant extract concentration and the best fit line was used to derive the estimated IC_50_ value from the concentration that could provide 50% of cell survival.

The concentrations of plant extracts giving 50% inhibitory concentration (IC_50_) were determined from three separate experiments. The IC_50_ of each plant extracts were then used as the treated concentration at 0 and 3 days against Kato-III and NUGC-4, which were assessed for apoptosis using a transmission electron microscopy (TEM). The assay was conducted in triplicate for each sample concentration from 3 separated assays.

### Sample preparation for transmission electron microscopy

Kato-III cells (1x10^6^ cells) and NUGC-4 cells (1x10^6^ cells) treated with each plant extract as well as the negative control (untreated cultures), were performed separately. Briefly, they were rinsed with D-Hank’s solution (Life technologies, Bangkok, Thailand) twice, and delivered into centrifuge tubes with a plastic scraper, followed by centrifugation at 2000 rpm for 15 min, with the supernatant removed. The precipitate was fixed in a solution containing 4% glutaraldehyde (Electron Microscopy Sciences, Bangkok, Thailand) and 2% paraformaldehyde (Electron Microscopy Sciences, Bangkok, Thailand) in 0.1 M phosphate buffer saline (PBS), pH 7.4, at 4°C for 1 h, then washed with 0.1 M PBS to remove the fixative. Specimens were postfixed in 1% osmium tetroxide (Electron Microscopy Sciences, Bangkok, Thailand) in the same buffer for 30 min, and dehydrated in a graded ethanol series for 10 min each. They were then cleared with two changes of propylene oxide and immersed in sequential mixtures of propylene oxide and Araldite 502 resin (Sigma-Aldrich Pte-Ltd, Singapore), at ratios of 3:1, 2:1, 1:2, and finally embedded in pure Araldite. Sections of 1 μm were cut using a MT-2 Porter-Blum ultramicrotome. The sections were subsequently mounted on copper grids, air dried and contrasted sequentially with 2% uranyl acetate (Electron Microscopy Sciences, Bangkok, Thailand) in 7% alcohol in the dark, and then treated with lead citrate (Electron Microscopy Sciences, Bangkok, Thailand). They were examined under a Philips CM 100 transmission electron microscope operating at 80 kV.

### Statistical analysis

Results were expressed as means±SD of replicates from 3 separated assays. Comparison between data sets was performed using one way analysis of variance (ANOVA) followed by Student’s t-test. All statistical analyses were performed using SPSS19. Differences were accepted as statistically significant at p<0.05.

## Results

### Total phenolic contents of plants extracts

Three edible folk plants from Northeastern region of Thailand (*S. gratum*, *J. gangetica* and *L. flava*) were extracted and their total phenolic content determined with the results shown in Table 2. Among these plant extracts, the highest level of total phenolic content was detected in *S. gratum* at 149.789 ±0.381 mg GAE/g. It was 10 folds significantly greater in content than that was identified in *J. gangetica* and *L. flava* (16.513 ±0.130 and 14.334 ±0.463 mg GAE/g, respectively, *p*<0.05).

**Table 2:** Mean total phenolic content of plant extracts expressed as GAE and anti-scavenging activity of the plant extracts represented as TEAC

**Plant extracts**	**Total plenolics(mg GAE/g dry weight)**	**Anti-scavenging activity (mM TEAC/g dry weight)**
*S. gratum* (Wight)	149.789 ± 0.381	2,823.521 ± 27.521
*J. gangetica L*.	16.513 ± 0.130^a^	313.141 ± 39.713^a^
*L. flava (L.)*	14.334 ± 0.463^a^	900.845 ± 20.346^a,b^

Values were expressed as means ± SD of replicates from 3 separated assays. ^a^*p*<0.05 VS *S. gratum*, ^b^*p*<0.05 VS *J. gangetica.*

### Antioxidant capacities of plant extracts

Antioxidant activities of ethyl acetate extracted of *S. gratum*, *J. gangetica* and *L. flava* are shown in Table 2. TEAC equivalent values for these plants were significantly different in descending order from *S. gratum* >*L. flava* >*J. gangetica* (2,823.521 ± 27.521, 900.845 ± 20.346, 313.141 ± 39.713, respectively, *p*<0.05). Noticeably, around 3–9 folds higher antioxidant activity of *S. gratum* was found compared with the other two species extracts. These were correlated well with total phenolic contents. (Correlation coefficient of R^2^= 0.935, Y=16.64x + 324.5).

### Cell growth inhibition

**Figure 1:**
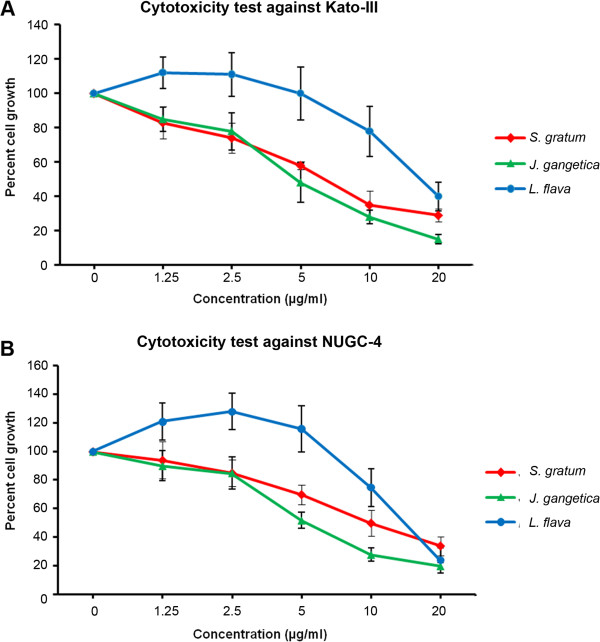
**Dose response studies of the plant extracts on two gastric cancer cell lines: (A) Kato-III and (B) NUGC-4.** The cells were treated with various concentrations (0, 1.25, 2.5, 5, 10 and 20 μg/mL) of *S. gratum*, *J. gangetica* and *L. flava* for 72 h. The antiproliferative effect was measured by MTT assay. Results were expressed as the means ±SD from three independent experiments.

**Figure 2:**
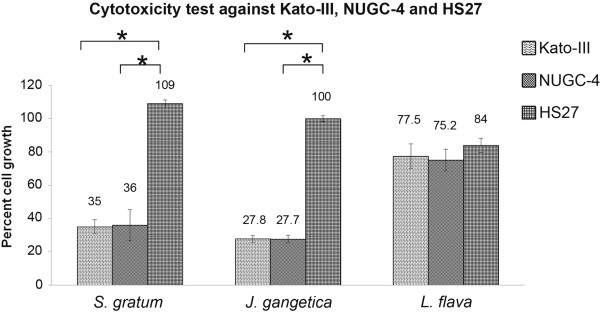
**Cytotoxicity test against Kato-III, NUGC-4 and Hs-27 after incubated with 10 μg/mL ethyl acetate extracted of *S. gratum*, *J. gangetica *and *L. flava.*** The antiproliferative effect was measured by MTT assay. Results were expressed as the means ±SD from three independent experiments. The results showed both *S. gratum* and *J. gangetica* strongly inhibited up to 70% gastric cancer cell growth while not destroying normal fibroblast cells Hs 27 (significant difference [*p<0.05]). This contrasted by the effect by *L. flava*, which demonstrated a diminished amount of cell growth, not only in gastric cancer cells but also on normal fibroblast cells as well.

Gastric cancer cell lines Kato-III and NUGC-4 and the human fibroblast cell line (control) were exposed to each plant extract (serial dilution concentration [1.25, 2.5, 5, 10 and 20 μg/mL]), to determine the growth inhibitory activity effect induced from each plant. After 72 h, viable cells were measured by MTT assay. Kato-III and NUGC-4 cells exposed to *S. gratum* and *J. gangetica* extracts resulted in a significant decrease in viable cells in a dose-dependent manner (Figure 1). At 20 μg/mL they all induced over 50% cell death in both gastric cancer cell lines. However, at 10 μg/mL the extracts produced from only *S. gratum* and *J. gangetica* demonstrated significant potent cytotoxicity (*p*<0.05) to induce over 70% cell death in Kato-III and NUGC-4 when compare with *L. flava* (Figure 2). In addition, these two plant extracts showed no effect on normal human foreskin fibroblast cell line (Figure 2). In contrast, *L. flava*’s effects diminished. Resulting in around 25% of cell death, with no significant difference between gastric cancer cells and normal fibroblast cell (Figure 2).

**Figure 3:**
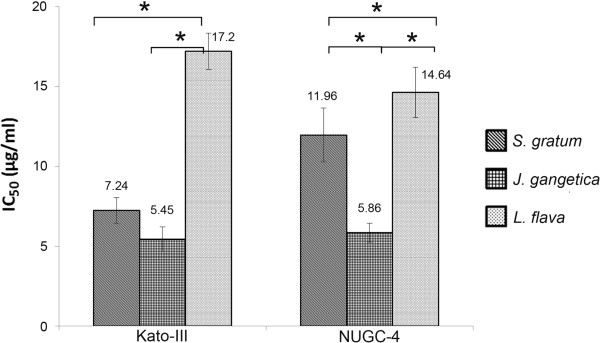
**Comparative cytotoxicity of IC**_**50 **_**of *S. gratum*, *J. gangetica *and *L. flava *on Kato-III, NUGC-4 and Hs27 by MTT assay.** Results were expressed as the means ±SD from three independent experiments (*p<0.05) and compared to Hs27-treated cells. *J. gangetica* showed the lowest IC_50_, whereas the highest was from *L. flava.* Significant differences (p<0.05) between *S. gratum* and *L. flava* were observed between both cancer cell lines.

The IC_50_ (μg/mL) values are summarized in Figure 3. The *J. gangetica* extract had the lowest IC_50_ values of 5.45 μg/mL and 5.86 μg/mL for Kato-III and NUGC-4, respectively. Similarly, the *S. gratum* extract showed higher cytotoxicity to the cancer cell lines with IC_50_ values in the 7.24 μg/mL – 11.96 μg/mL range, whereas, the highest IC_50_ was from *L. flava* extract 17.20 μg/mL and 14.64 μg/mL for Kato-III and NUGC-4 respectively. This was significantly different (*p*<0.05) when compared to the other two plant extracts (Figure 3).

### Ultrastructure alterations of Kato-III and NUGC-4 cell lines induced by *S. gratum, J. gangetica* and *L. flava*

**Figure 4:**
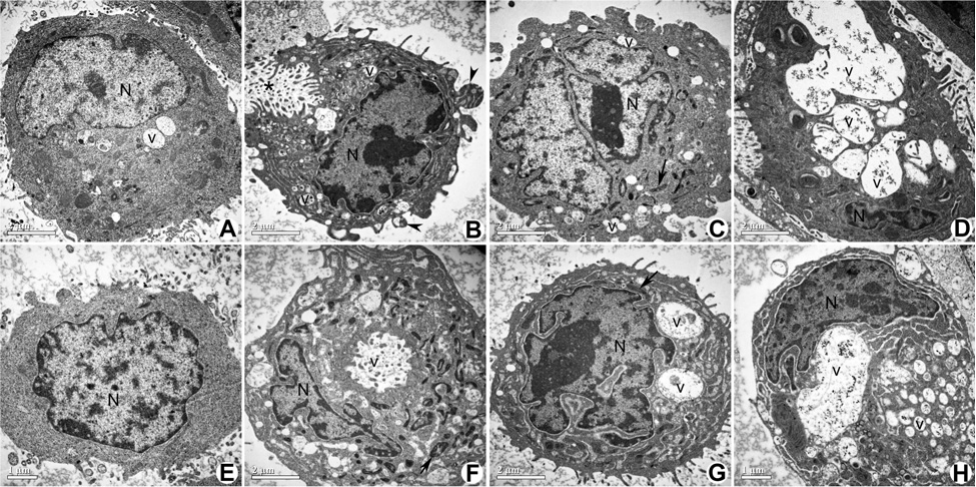
**Electron micrographs comparing the effects of *S. gratum*, *J. gangetica *and *L. flava *on Kato-III (A-D) and NUGC-4 (E-H) 3 days post treatment.** Scale bar 2 μm. **A**) Untreated Kato-III cell shows very few vesicles (v), a rather uniform rounded shape and chromatin scattered throughout nucleus (N). **B**) *S. gratum* treated Kato-III cell shows condensed chromatin in nucleus (N), apoptotic body formation (arrowhead) and many vesicles. The cell is dispersing as granular debris (*). **C**) Kato-III cell treated with *J. gangetica*. This cell shows chromatin condensation around the periphery of the nucleus, membrane bound organelles (arrow) and numerous vacuoles (v). **D**) Kato-III cell after exposure to *L. flava* shows a large number of autophagic vacuoles (v), and a shrinking nucleus (N) with condensed chromatin. **E**) Untreated NUGC-4 cell shows no condensation of chromatin in nucleus (N) and a rather uniform rounded shape. **F**) NUGC-4 cell treated with *S. gratum* shows chromatin condensation nucleus, numerous vesicles (v) and many membrane bound organelles (arrow). **G**) NUGC-4 cell after exposure to *J. gangetica* shows a nucleus condensed chromatin, membrane bound organelle (arrow) and vesicles (v). **H**) Morphological changes observed in *L. flava* treated NUGC-4 cell were comprised with chromatin condensation in the nucleus (N), and many heterogenous vesicles of varying size (v).

In order to determine whether the growth inhibition by plant extracts were associated with apoptosis, we further examined the morphological changes of Kato-III and NUGC-4 gastric cancer cell lines under transmission electron microscope. The control cells nuclear structures appeared intact (Figure 4A: Kato-III, E: NUGC-4), while the cells treated with those of the plant extracts demonstrated ultrastructural changes in several manners (Figures 4B-4D: Kato-III, and 4 F-4H: NUGC-4, respectively). In detail, the Kato-III cells treated with *S. gratum* displayed a condensed nucleus with chromatin condensation, apoptotic body formation (Figure 4B), and dispersing granular debris. While for Kato-III cells treated *J. gangetica*, these displayed chromatin condensation, membrane bound apoptotic bodies and numerous vesicles (Figure 4C). Whereas the morphological changes found in *L. flava* treated Kato-III cells, displayed shrunken nucleus with chromatin condensation and numerous heterogenous vesicles, including extensive features of intracellular vacuolization (Figure 4D).

*S. gratum* treated NUGC-4 cell lines exhibited apoptosis with compacting nucleus and production of membrane bound apoptotic bodies and numerous vesicles (Figure 4F). However in comparison, NUGC-4 cells treated with *J. gangetica*, produced early stages of apoptosis with chromatin condensation and numerous vesicles (Figure 4G). While cells treated with *L. flava* showed peripheral chromatin condensation nucleus with numerous heterogenous vesicles and blebbing (Figure 4H).

## Discussion

Serendipitous observations have shown that plants, traditional herbs and teas can be harnessed to potentially win the fight in battling cancer; a worldwide health problem. However, it is not until these phytochemicals are tested *in vitro* and *in vivo* that we can know for sure how far they can go in keeping this disease under control [31-34]. In Thailand gastric cancer is a scourge, however the unusually lower gastric cancer incidences in the Northeastern part of Thailand is of considerable interest. The fact that *S. gratum*, *J. gangetica* and *L. flava* are indigenous to the area and form a major part of the routine dietary supplement in the local population, we therefore decided to investigate whether these folk plants are potential candidates for the safe and reliable control of gastric cancer. Though there are many reports to clarify their anti-oxidant activities, this study provides the first evidence of their potent cytotoxic effects and apoptotic induction based on ultrastrutural characteristic on gastric cancer.

These plants were firstly extracted with ethyl acetate and then analyzed for their phenolic contents using Folin-Ciocalteu method. The ethyl acetate extract of *S. gratum* demonstrated that it possessed water and ethanolic extracts strongly correlating to those aqueous extracts found by Senggunprai *et al.*, although in significantly lesser amounts [22]. This suggests that the potent compounds to battle with gastric cancer cell lines in *S. gratum* exist primarily in medium polarity. Also in agreement with findings reported by Ruan *et al.*, whom indicated that the active components of *S. cumini* (a plant within the Myrtaceae family similar to *S. gratum*), are mainly in the medium-polarity ethyl acetate fraction [35]. However, even if none of the major constituents of *S. gratum* have been determined chemically, they are proven to prevent not only oxidative damage but also know to alleviate nitrosative stresses [23]. Our data therefore strengthens the previous research in this field that *S. gratum* has strong antioxidant activity, and therefore provides further support for considering *S. gratum* as a food supplement for the people of Thailand.

In addition, some Thai plants are reported to possess antimutagenic properties and activities related to inflammation; key properties of many antioxidants [36,37]. To measure anti-oxidative potential, ABTS and DPPH assays are favourable because of their relative ease of use and their ability to yield reliable results [38]. We, therefore, determined antioxidant activity of the plant extracts to reflect the counteract properties to the mutagen in these plants by the ABTS assay. Moreover since it has been recommended to reduce the background interference originating from plant extracts at a wavelength of 740 nm [39]. From our data we can observe a significant positive correlation between total phenolic contents and antioxidant activity of those three plant extracts (R^2^= 0.935, Y=16.64x + 324.5), consistent with other studies [40,41]. Of all three plants investigated, *S. gratum* had the highest level of phenolic contents, as well as the strongest antioxidant capacity, which correlates to and is supported also by studies headed by Maisuthisakul [42]. This group showed that the antiradical activity (1.8 L/EC_50_), total phenolic (57.3 mg GAE/g db) and total flavanoid (23.6 mg RE/g db) of *S. gratum* was not only reflective of our findings, but their additional experiments that explored the antioxidant capacity in plasma of β-thalassemia/Hb E patients treated with *S. gratum*. This overall places further emphasis that *S. gratum* is an excellent candidate for gastric cancer treatment, even with short term intake [43]. Moreover, leaf extracts of *S. gratum* which has proven radical scavenging activity and anti-inflammatory properties, gives more weight to this conviction [23].

Additionally, aberrant cell survival has always been closely investigated to perhaps find a strategy to control their proliferation, which often results from inhibition of apoptosis, leading to tumor progression and oncogenesis. It is clear that cancer cells often gain a selective growth advantage by blocking apoptosis [44]. Therefore, as the induction of apoptotic cell death is an important mechanism in anticancer properties in many anticancer drugs [45,46], this is the first study to further examine whether or not, *S. gratum*, *J. gangetica* and *L. flava* induce apoptosis (cell death) in gastric cancer cells. Following on from our initial cytotoxicity trials, *S. gratum* and *J. gangetica* were found to be better dose-dependently than *L. flava* through the higher inhibition of cell viability in Kato-III and NUGC-4, whereas they had almost no effect on the growth of normal fibroblast cells Hs27. Before continuing with our morphological investigation, ethanolic extracts of those three plants were also analyzed for total phenolic content, and additionally evaluated for their cytotoxicity against gastric cancer cell lines. The results showed that they had only a marginal 20% capacity to inhibit the growth of gastric cancer cell lines (data not shown). This finding thus directed us to conduct on-going research towards using ethyl acetate extracts. Therefore based on this information and our cytotoxicity data, which gave a more favorable and clearer response, we therefore continued with ethyl acetate extracts of *S. gratum, J. gangetica* and *L. flava*, and photo documented the characteristic morphological changes of each cell line under TEM. At 72 h post treatment, *S. gratum* and *J. gangetica* produced ultrastructural alteration of chromatin aggregation, mitochondrial denaturation and apoptotic body formation, as well as cytoplasmic compartments, swelling and disappearance of mitochondrial cristae in Kato-III and NUGC-4. These features are consistent to those commonly found in apoptotic cells, which by comparison, closely reflects as a dose-dependent way of cimetidine induced apoptosis in human gastric cancer cells SGC-7901 and MGC-803 [13]. Moreover, the similar morphologic features of apoptosis in gastric cancer cells SGC-7901 treated with tributyrin (i.e. not limited to chromatin aggregation, the unravelling of mitochondria, cellular breakdown and cytolysis) might be explained by the disturbance of apoptotic mechanism including the down-regulation of Bcl-2 expression and the up-regulation of Bax expression as demonstrated by Yan and Xu [14]. In the more recent investigations of SGC-7901 and MGC-803 cells treated with cimetidine, an increase in Bax/Bcl-2 ratios and activation of caspases were also observed [13]. These proteins are members of the Bcl-2 family which are important regulators in the apoptotic pathway that can suppress Bcl-2 amd Bcl-xl activity, or promote Bax upregulation, and thus apoptosis. Moreover, when we consider the anti-cancer effects of megastigmane glycoside, the major compound in *J. gangetica*, it has been reported that apoptotic induction can be achieved in human melanoma cell lines through inhibiting NF-κB activation [47]. So, the phenolic extracts produced by *S. gratum* and *J. gangetica* should be investigated further to assess their full function.

This study demonstrates that *S. gratum* and *J. gangetica* can be considered candidate anti-carcinogenic agents, adding to previous reports demonstrating that health benefits directly come from synergistic combinations of the phytochemicals compounds existing in each plant [48]. Therefore further studies are now needed to focus on their function on gastric cancer gene expression pathways, verify the individual active components of each plant, and to determine their extract chemical structures. Development of extension projects in this area will bridge a gap and create new knowledge to alleviate the detrimental effects of this disease.

## Conclusions

In summary, among the three local edible plants analyzed in this study, our present findings indicate that *S. gratum* and *J. gangetica* significantly inhibited growth of human gastric cancer cells, Kato-III and NUGC-4. These two plants also were effective in inducing cellular alterations, determined by characteristic ultrastructural changes which could reflect cytotoxic activity. These two plants hold the highest levels of total phenolic contents, and demonstrate strong antioxidant activity. When considering their potential and safety, we can move closer to identifying novel candidates not only for the treatment of gastric cancer by determining their effect in the therapeutic agents, but also by promoting the Thai people to include these plants as a daily supplement for the prevention of the disease. We are now moving forward to breakdown and assess the individual plant components of *S. gratum* and *J. gangetica*, and their efficacy against gastric cancer cells.

## Competing interests

The authors declare that they have no competing interests.

## Authors’ contributions

PS and PR contributed in the experimental design and carried out the experiments, analyzed and interpreted the data, and contributed in drafting and revision the manuscript. PB carried out the plant extraction and SP carried out some of the experiments. All of the authors read the manuscript, contributed in correcting it and approving its final version.

## Pre-publication history

The pre-publication history for this paper can be accessed here:

http://www.biomedcentral.com/1472-6882/13/60/prepub
